# Strategies Affording Prevascularized Cell-Based Constructs for Myocardial Tissue Engineering

**DOI:** 10.1155/2014/434169

**Published:** 2014-01-05

**Authors:** Claudio Muscari, Emanuele Giordano, Francesca Bonafè, Marco Govoni, Carlo Guarnieri

**Affiliations:** ^1^Department of Biomedical and Neuromotor Sciences (DIBINEM), University of Bologna, Via Irnerio 48, 40126 Bologna, Italy; ^2^BioEngLab, Health Science and Technology, Interdepartmental Center for Industrial Research (HST-CIRI), University of Bologna, 40064 Ozzano Emilia, Italy; ^3^Laboratory of Cellular and Molecular Engineering “Silvio Cavalcanti,” DEI, University of Bologna, 47521 Cesena, Italy

## Abstract

The production of a functional cardiac tissue to be transplanted in the injured area of the infarcted myocardium represents a challenge for regenerative medicine. Most cell-based grafts are unviable because of inadequate perfusion; therefore, prevascularization might be a suitable approach for myocardial tissue engineering. To this aim, cells with a differentiation potential towards vascular and cardiac muscle phenotypes have been cocultured in 2D or 3D appropriate scaffolds. In addition to these basic approaches, more sophisticated strategies have been followed employing mixed-cell sheets, microvascular modules, and inosculation from vascular explants. Technologies exerting spatial control of vascular cells, such as topographical surface roughening and ordered patterning, represent other ways to drive scaffold vascularization. Finally, microfluidic devices and bioreactors exerting mechanical stress have also been employed for high-throughput scaling-up production in order to accelerate muscle differentiation and speeding the endothelialization process. Future research should address issues such as how to optimize cells, biomaterials, and biochemical components to improve the vascular integration of the construct within the cardiac wall, satisfying the metabolic and functional needs of the myocardial tissue.

## 1. Introduction

One of the most ambitious objectives for cardiac tissue engineering aimed at repairing an infarcted cardiac region is the production of a stem cell-based functional tissue that can be fully integrated within native myocardium [1–3]. However, although basic researches and preclinical studies have paved the way for translational applications of cell therapy for cardiac repair, so far clinical trials have failed to reproduce relevant positive results [4]. Ongoing efforts are thus needed to optimize most steps involved with a therapeutic approach to myocardial infarction and failure using stem cells, including cell preparations, cell delivery techniques, and cell survival [5].

Up to now, stem cells transplanted alone or carried onboard of polymeric devices have shown a short survival in the heart because these grafts were not adequately perfused by coronary vessels [6, 7]; hence, most grafted cells rapidly die or migrate near the border zone of the infarcted area, close to the coronary vessels [8]. To provide sufficient oxygen and nutrients for survival, metabolically active grafts should be supplied by a vascular network reaching cardiomyocytes within a 150–200 *μ*m distance [9]. Therefore, the laboratory realization of prevascularized tissues represents a stimulating chance for myocardial tissue engineering, although this approach can solve only one of the several factors at play in such a complex system [7]. In fact, to be successfully grafted, a cardiac construct must (i) be sufficiently thick to contract with the appropriate strength and show the same compliance of the cardiac wall, (ii) beat synchronously with the neighboring cardiomyocytes, (iii) not generate inflammatory reactions, (iv) produce a suitable extracellular matrix, and, ultimately, (v) improve cardiac function in a relevant manner. Other concerns are inherent to the scaffolds and the cells intended for the purpose. Natural fibrous biomaterial, for example, collagen or hyaluronan, cannot be easily produced in a scalable manner, while artificial solid scaffolds are usually quite stiff to properly support the systodiastolic dynamic. When applied on the epicardial surface, solid patches can hardly integrate with the underlying infarcted tissue which is often fibrous and poorly perfused. On the other hand, gelled substrates with the cells they convey are largely rejected during their injection because of the lack of space in the compact cardiac wall. Ideal scaffolds should also show additional properties, including the lack of toxicity and immunogenicity, the presence of a favorable microenvironment—such as the appropriate cell adhesion molecules—for cell proliferation and differentiation, an adequate porosity allowing cell and vascular connections, and a bioresorption rate fitting with cardiac matrix turnover. Inasmuch as the cellular counterpart is concerned, terminally differentiated cells, such as cardiomyocytes and vascular endothelial cells, cannot be a suitable graft because they do not survive any longer after *in vivo* transplantation. On the other hand, more viable and proliferating cells, such as stem cells and progenitor cells, are not yet proven to differentiate into cardiomyocytes that fully accomplish the electromechanical needs of a functional heart. Furthermore, most allogeneic cells are rejected, while those derived from pluripotent cells could be tumorigenic [10].

Although some of these concerns are not solved by construct prevascularization, viability, permanence, proliferation, and differentiation of the transplanted cells should ameliorate together with the overall process of cardiac regeneration.

Up-to-date reviews deal with cells and/or scaffolds suitable for vascularizing engineered tissues as a general process. Here, in a more specific manner, we will focus on myocardial regeneration and discuss the proangiogenic properties of cardiac patches that are useful to survive in the hostile environment of an ischemic heart, commenting also on the most prominent techniques used for their production (Table 1).

## 2. Selected Cells and Scaffolds to Create Cardiac Constructs

Extensive investigations have been devoted to define cells suitable for supporting the growth of new vessels and also providing significant restoration of coronary blood perfusion in an infarcted cardiac region [27, 28]. In this regard, cells can be substantially classified according to their main role in the vascularization process, since some of them can directly participate in the formation of new vessels or stimulate the process by releasing paracrine factors. However, a clear delimitation between these two categories is not always easily drawn because most cells can exert both functions. Endothelial progenitor (precursor) cells (EPCs) are the most promising among those contributing to the vessel structure because they can survive and proliferate better than terminally differentiated endothelial cells (ECs), promoting both angiogenesis and vasculogenesis. Moreover, EPCs are able to release several growth factors useful for improving neovascularization [29, 30].

However, experimental and clinical trials have not shown important benefits from their transplantation, and their uncertain effects are probably a consequence of the low number of EPCs that can be expanded *ex vivo* and then transferred into the ischemic tissue [31]. A similar contribution to the formation of a vessel wall has been described for adipose-derived stem (stromal) cells (ADSCs) just after their isolation [32, 33]. These cells belong to the wide family of mesenchymal stem cells (MSCs) that can be originated from different biological sources. Indeed, besides adipose tissue, virtually any kind of postnatal tissue deriving from embryonic mesenchyme appears as the site of resident MSCs [34, 35], bone marrow being the most exploited source [36]. MSCs can differentiate into cardiomyocyte precursor cells [37, 38] and into smooth muscle cells or other cells supporting the architectures of the vascular wall [35]. Furthermore, they release angiogenic factors, such as the vascular endothelial growth factor (VEGF), hepatocyte growth factor (HGF), and basic fibroblast growth factor (bFGF), to recruit and stimulate other resident and circulating cells involved in vessel formation. Most of these angiogenic growth factors exert also protective effects and increase cell survival [39, 40]. In addition to a sustained angiogenic stimulation, the viability of grafted cells might be enhanced by means of strategies that increase their resistance to a condition of underperfusion. As an example, cell preconditioning through hypoxic pretreatments has been performed before cell transplantation [41]. Hypoxic preconditioning has also been invocated as a natural process that allows stem cells to survive in microenvironments with a low oxygen tension [42].

Cells differentiating into beating cardiomyocytes, including embryonic stem cells (ESCs) [43], induced pluripotent stem cells (iPSCs) [44], and resident cardiac stem cells (CSCs) [45], represent the muscle counterpart of the prevascularized constructs.

Bioactive scaffolds employed as a support for engineered tissue endothelialization are fabricated with synthetic or natural polymers and possess several properties useful for facilitating neovascularization [46]. Favorable materials might be also biofunctionalized in order to accelerate *in situ* endothelialization and provide a specific microenvironment mimicking the natural properties of the native tissue [47]. Examples of molecules that can be conjugated to the polymer material aimed at improving vessel formation are usually natural components of the extracellular matrix or their functional domains. Moreover, many chemoattracting factors belonging to the families of growth factors and cytokines can be bound to the polymer fibers to recruit provascularizing cells from blood and neighboring tissues [48].

## 3. Prevascularized Cardiac Patches 

### 3.1. Basic Constructs

3D tissue-engineered cardiac muscle constructs, aimed at the promotion of a functional vascularization using two types of cells, ECs and muscle cells, have been widely investigated [48]. More recently, an interesting study exploited triculture cell constructs originated by human ESC-derived cardiomyocytes, human umbilical vein endothelial cells (HUVECs), and mouse embryonic fibroblasts (MEFs), the latter showing properties similar to MSCs [11]. These cells were seeded onto a biodegradable porous scaffold and the resulting vascularized construct was transplanted in a rat heart. The number of new patent blood vessels was significantly greater in the hearts engrafted with the tri-culture construct than in those containing only the ESC-derived cardiomyocytes. Immunostaining for CD31 and *α*-smooth muscle actin (*α*-SMA) demonstrated the formation of both donor and host-derived vasculature within the engrafted tri-culture tissue constructs. Similarly, Stevens et al. [12] differentiated human ESCs into cardiomyocytes by using activin A and bone morphogenetic protein 4 (BMP4) and then placed them, in suspension, within a rotating orbital shaker to create a scaffold-free vascularized cardiac tissue cocultured with HUVECs, or human ESC-derived endothelial cells, and MEFs. These constructs actively contracted *ex vivo* in response to electrical pacing, up to frequencies of 2-3 Hz, and formed a vascularized myocardium in nude rat hearts. The patch was engrafted in the heart by removing part of the pericardium. The epicardium was than scuffed slightly with a cotton swab and a single patch was placed or sutured directly onto the heart. More clinical relevant patches composed entirely of human cells were then obtained by the same authors changing MEFs with human dermal fibroblasts. This human tri-culture was again able to form viable myocardium and to integrate human coronary microvessels in the rodent heart. This study demonstrates the importance of including vascular and stromal components in a cardiac patch, although it does not give conclusive information about its behavior in an infarcted heart.

### 3.2. Microvascular Modules

Using HUVECs as endothelial cell source, microvascular segments stabilized in collagen were produced and transplanted into the ischemic mouse myocardium [13]. Modules were prepared using collagen gelled in sterile polyethylene tubing which was cut into small pieces (2 mm long–0.6 mm diameter). HUVECs were seeded dynamically onto the module surface and incubated for 7 days before implantation. These patches formed *in vivo* well-vascularized structures and, although devoid of cardiomyocytes, they were also able to improve cardiac contractility. Microvascular modules might be grafted at first to serve as vascularized platforms for the subsequent transplantation of contractile cardiac patches in order to complete the repair process.

### 3.3. Inosculation from Vascular Explants

A further progress in the efficiency of scaffold vascularization was obtained using living organs as donors of sprouting vessels. Neonatal rat cardiac cell patches containing angiogenic factors into an alginate scaffold were prevascularized for one week in the rat omentum and subsequently transplanted into the infarcted rat cardiac region by a single stitch seven days after the induction of the myocardial infarction [14]. The effects of transplantation went towards the formation of new vessels, and the electromechanical function of the heart was also improved.

With a similar strategy, a capillary network was generated *ex vivo* from vascular explants on a micropatterned polydimethylsiloxane substrate with grooves of different (25, 50, 100 *μ*m) sizes [15]. This prototype consisted of two branching vessels, an artery and a vein, that formed a microvascular bed suitable for *in vitro* rapid vascularization of engineered tissues. Moreover, the controlled release of thymosin *β*4 (T*β*4) from the coating of the substrate promoted the formation of a vascular network. T*β*4, an angiogenic and cardioprotective peptide, has been described to enhance neonatal rat cardiomyocyte survival by inducing coronary vascularization and up-regulation of Akt activity [49]. In particular, T*β*4 initiates angiogenesis and sustains vascular stability because of its ability to recruit endothelial and smooth muscle cells. The capillary outgrowth, connected between parent explants obtained from animal or human vessels, was further accelerated by the application of soluble VEGF and HGF. The cells lining the lumens of the tubules expressed markers of mature endothelial cells such as CD31, VE-cadherin, and von Willebrand factor. Cardiac tissues engineered around the resulting vasculature exhibited improved functional properties, cell striations, and cell-cell junctions, when compared with tissues without prevascularization.

### 3.4. Mixed Cell Sheets

Cell sheet technology exploits a scaffold-free process of alternatively stacked monolayers of cells. One of the main advantages to use cell sheets with respect to scaffold-supported cells is that the former can more easily communicate electrically via gap junction formation. To improve and speed the vascularization of a cardiac graft, mixed cell sheets of endothelial cells and cardiomyocytes have been used. Sasagawa et al. [16] obtained a pre-vascularized three-dimensional cell-dense tissue by means of a particular cell-sheet manipulation technology. HUVECs sandwiched between sheets of human skeletal myoblasts sprouted a capillary-like network. After transplantation into the subcutaneous tissues of nude rats, the preformed endothelial network of a five-layer myoblast sheet construct was efficiently connected to the host vessels and allowed the graft to be well perfused.

## 4. Technologies Exerting Spatial Control over Vascular Cells

To drive a spatial order over the placement and organization of individual cells in 3D scaffolds, self-assembling organ printing [50] and lithographic fabrication [15, 17] of vascular-inductive matrices have been proposed. A system for scaffold-guided tubulogenesis coupled with 3D organotypic culture was recently created to guide microvessel formation [18]. In this study, using photolithography, rigid and soft scaffolds were fabricated by introducing light curing epoxy and polydimethylsiloxane stamps directly into collagen. The scaffolds were then seeded with dermal microvascular ECs and placed between the gelled layers of collagen containing dermal fibroblasts. This cell system was then cultured for up to two weeks to allow vessel maturation. Morphological analysis of thin tissue sections confirmed vessel formation along the microchannel walls with both scaffolds, showing the formation of a natural architecture of ECs, fibroblasts, and basement membranes.

Using a computerized carbon dioxide laser, Marsano et al. [19] created cubically packed channels in a porous poly(glycerolsebacate) elastomeric scaffold where neonatal rat cardiomyocytes and transduced myoblasts releasing VEGF formed a cardiac tissue with geometrically ordered microvessels. This patch was sutured onto the infarct region one week after ischemia in a mouse model of myocardial infarction. Ten weeks later, the vascular ingrowth into the patch area was greatly enhanced as well as the cardiac contractility.

A natural alternative to artificially micropatterned scaffolds for the realization of a hierarchical distribution of the vasculature is represented by the decellularized cardiac extracellular matrix (ECM). The cellular ECM isolated from the heart offers indeed several advantages which are not only limited to the excellent biomechanical properties of the constituent fibers but also include the preservation of the 3D architecture of the coronary bed. Usually, after decellularization, the vascular network is maintained to the first three-four branches from the main coronary vessels. Hearts from rats and pigs have been successfully decellularized and reseeded with various types of cells including vascular endothelial cells and stem cells [20]. These constructs can be investigated as biomedical patch for transmural transplantation strategies or for screening new therapeutic agents in handily models of cardiac ECM.

## 5. Bioreactors Accelerating *Ex Vivo* Endothelialization of Solid Scaffolds

Most techniques generating endothelial-lined tubule-like structures into solid scaffold use microfluidic devices for high-throughput production [21]. Bioreactors exerting mechanical stress have been also employed in order to simultaneously improve muscle differentiation and create in the medium a turbulence useful for speeding the endothelialization process [22, 51].

### 5.1. Bioreactors Inducing Shear Stress

A general feature of perfusion bioreactors, that is, the application of a flow by forcing the medium into the entire cell-seeded scaffolds, enables efficient mass transfer of oxygen and other soluble factors. In addition, the perfused medium creates a frictional force on the surface of the cells, termed shear stress, that can influence cell behavior according to the phenotype. Vascular ECs are normally exposed to high fluid shear stress, since they constitute the inner layer of the arterial wall. Shear stress is an important regulator of the fate of ECs and EPCs, as demonstrated by the related changes in their metabolic, differentiating, and functional properties. Most studies investigating the effects of shear stress on cultured cells have been conducted in devices that enabled the monitoring of one shear rate at a time. However, multi-shear microdevices have been more recently designed for both 2D and 3D cultured cells. In particular, microfabricated perfusion bioreactors have been developed to allow an *in vitro* 3D cell culture with high cell density similar to that found in native organs. Similarly, a microfabricated multishear perfusion bioreactor was designed to deliver different levels of shear stress to six constructs simultaneously during a single run [23]. HUVECs, seeded in alginate scaffolds within the bioreactor and subjected to different levels of shear stress for 24 hours, responded accordingly by expressing the intercellular adhesion molecule 1 (ICAM-1) and the phosphorylated isoform of the endothelial nitric oxide synthase (eNOS).

Researchers intending to create functional vascular networks with channel patency should also take into account that the inner surface must be antithrombogenic. In order to develop a rapid endothelialization for creating an antithrombogenic surface mimicking the natural vessel wall in an artificial vascular network, Kang at al. [24] investigated how shear stress preconditioning modified the surface of a polycaprolactone-based scaffold. To provide cells with physiological shear stress conditions, the perfusion bioreactor was composed of a peristaltic pump, a medium reservoir, a gas exchanger, and a perfusion chamber designed to simultaneously load six scaffolds. The increase in shear stress caused a rapid proliferation of the mouse ECs used to cover the polymer surface. The DNA content increased by 91% within 12 hours after exposure to higher shear stress, as well as VE-cadherin and PECAM. These effects were further improved using bioactive materials such as collagen and recombinant adhesive protein fused with arginine-glycine-aspartic acid peptide (RGD). Platelet adhesion tests demonstrated that the RGD-functionalized scaffold exerted an anti-thrombogenic potential and that it was increased after endothelialization.

### 5.2. Pulsatile Flow Bioreactors

Other studies employing bioreactors focused on cell sheet technology in order to engineer cardiac constructs with perfusable blood vessels [52]. Sekine et al. [25] overlaid a triple-layer of mixed neonatal rat cardiac cell sheets with ECs on the resection of a rat femoral muscle connected through its artery and vein to a bioreactor perfusing the ongoing construct. At first, the number of these layers was limited to three because it represented the thickness limit on culture dishes without blood vessels. The femoral vessels were connected to two thin polyurethane tubes for the culture medium perfusion, and the overlaid cocultured sheets formed new blood vessels connected with those originated from the vascular bed. The flow was generated by a delivery pump from the media reservoir monitored by a pressure sensor positioned on the arterial inlet side, passing through the tissue culture chamber and flowing into a waste fluid tank. Co-cultured ECs significantly contributed to increase blood vessel tubular structure formation within the cell sheet constructs. Moreover, the addition of bFGF to the medium promoted perfusable blood vessel communication between the constructs and the vascular bed. To scale up the production of this bioengineered tissue, triple-layer cocultured cardiac cell sheets were repeatedly overlaid on the vascular bed and perfused using this bioreactor system. Each second triple-layer tissue was overlaid on the first one after three days of cultivation and the whole construct was perfused for further three days. This multiple-step procedure was followed up to the formation of 12 layers, producing thicker and more cell-dense cardiac tissues than the single-step counterpart. A six-layer tissue graft was then transplanted in the neck of a nude rat. The protruding femoral artery and vein of the graft were reconnected to the carotid artery and the jugular vein, respectively. This graft was maintained for two weeks and showed to be well perfused and to contract spontaneously.

### 5.3. Bioreactors Developing Mechanical Stress

The developing engineered heart not only requires an efficient blood supply but also must ensure an adequate mechanical load. A study on the effects of uniaxial cyclic mechanical stress on human cardiomyocytes, derived from ESCs and iPSCs grown in a 3D collagen matrix, demonstrated that this technique promoted a twofold increase in cardiomyocyte number size. Moreover, the physical stretch enhanced both myofibrillogenesis and sarcomeric banding and induced the alignement of collagen fiber bundles in the ECM [26]. Using this set-up, the addition of HUVECs further increased cardiomyocyte proliferation under stress conditions. These human cardiac constructs also generated Starling curves, increasing the active force in response to a parallel enhancement of resting length. Stromal supporting cells (MEF or human MSCs) added to the construct further augmented the formation of vessel-like structures tenfold. Moreover, when these cardiac patches were sutured onto the epicardium of athymic rats, they formed grafts perfused by human microvessels thanks to their connection to the host coronary network.

## 6. Limits to Transplantation of Prevascularized Constructs in an Infarcted Heart

The complexity of a method for delivery and connection of a cardiac tissue construct represents a major limit to the good integration and functionality of the graft. According to the type of pre-vascularization, the following main strategies have been adopted: (i) transplantation of microvascularized gelled material into the infarct region/border zone of the heart, (ii) suture/application of the patch on the infarcted tissue, (iii) insertion of the construct into a “pouch” created into the thickness of the cardiac wall [8, 28], and (iv) anastomosis of the inlet/outlet perfusion conduits of the construct, often described as arteriovenous loops, with branches of coronary vessels [53, 54].

All these methods represent a progress with respect to the transplantation of constructs which are not prevascularized, since they are doomed to die in a short time. However, although the inosculation of the prevascularized constructs from the underlying native cardiac tissue is accelerated, the anatomofunctional integration of the muscle counterpart requires much more time. Moreover, the inherent ischemic and/or fibrous features of the ischemic myocardium negatively affect the possibility to obtain a suitable graft. Only long-term animal models of cardiac infarction will be able to ascertain whether such strategies can really contrast the pathological remodeling of the cardiac wall and restore the electromechanical activity of the damaged heart.

## 7. Conclusions

Pre-vascularized engineered cardiac tissues rapidly connected to the vascular bed of the heart allow transplanted cells to survive, remain in the graft region, and better accomplish the differentiation/proliferation process and the anatomofunctional integration with neighboring cells. Basic constructs promote *in vivo* angiogenesis thanks to the presence of, for example, vascular cells, fibroblasts, and mesenchymal stem cells. Their effects can be reinforced by angiogenic growth factors conveyed by the scaffold itself or released by some cellular component particularly prone to exert this paracrine action. Although constructs engineered with vascular cells can be fabricated quite easily, they need a certain time to produce a vascular network when implanted in the heart. Microvascular 3D segments stabilized in a gelled biomaterial provide a preformed primordial conduit network which accelerates the integration between the construct itself and the native cardiac tissue. A more natural prevascularization of the construct can be obtained using explants sprouting microvessels. On the other hand, to overcome the problem of scaffold biocompatibility, the cell-sheet technology can be adopted and enriched in endothelial cells to stimulate angiogenesis. Moreover, multiple applications of mixed muscle-endothelial cell sheets can realize vascularized patches with adequate thickness for sustaining cardiac contractility. Finally, advanced technologies guiding high-throughput hierarchical vessel growth represent the ultimate frontier to scale up the production of suitable pre-vascularized constructs for clinical applications.

## Figures and Tables

**Table 1:** Strategies to promote functional vascularization of cardiac patches.

Cell source/vascular explants	Scaffold/cell sheets	Bioreactors/other devices	Biological effects	Ref.
hESC-derived cardiomyocytes /HUVECs/MEFs	Porous scaffold	None	Formation of both donor and host-derived vasculature within the engrafted triculture tissue constructs	[11]

hESC-derived cardiomyocytes /HUVECs/MEFs /human dermal fibroblasts	None	Rotating orbital shaker	Contraction of constructs in response to electrical pacing up to frequencies of 2-3 Hz/formation of new blood vessels in rat myocardium	[12]

HUVECs	Collagen	Intact microvascular segments gelled in polyethylene tubes	Formation of patent vascularized structures in the infarcted mouse heart/increase in cardiac contractility	[13]

Neonatal rat cardiac cells	Alginate plus angiogenic factors	Inosculation from heterotopic rat omentum of microvessels within the construct	Structural and electrical integration of cardiac patch into the infarcted rat myocardium/induction of thicker scars/prevention of chamber dilatation and ventricular dysfunction	[14]

Mouse cardiac tissues, thoracic artery, vena cava/rat and human arteries and veins/neonatal rat cardiomyocytes	Thymosin *β*4-releasing hydrogel scaffold	Polydimethylsiloxane substrate with grooves produced through soft lithography	*In vitro* improved functional properties of the cardiac construct/cell striations/cell-cell junctions/guided capillary outgrowths	[15]

HUVECs/human skeletal myoblasts	Cell sheets	HUVECs sandwiched between sheets of myoblasts	Cell sheets sprouted a capillary-like network and efficiently connected to the host vessels	[16]

HUVECs	Poly(ethylene glycol)-diacrylate hydrogel scaffold	Device micropatterned with cell-adhesive ligands by photolithography	Spatial regulation of the angiogenic response	[17]

Human microvascular endothelial cells/human dermal fibroblasts	Collagen	System for guided tubulogenesis coupled with 3D organotypic culture	Endothelial tube formation/endothelial vessels surrounded by collagen type IV	[18]

Neonatal rat cardiomyocytes/transduced mouse skeletal myoblasts releasing VEGF	Porous poly(glycerol sebacate) scaffold	Channels produced by a CO_2_ computerized laser	Formation of a geometrically ordered mature vascular network in mouse infarcted myocardium	[19]

Rat MSCs/HUVECs	Decellularized porcine heart tissue	None	Preservation of macro- and micro-vascular architecture and ultrastructure of native cardiac tissue	[20]

Human microvascular endothelial cells	Collagen or poly-D-lysine-hydrobromide	Microfluidic device	Gradients in channeled scaffolds create high-throughput angiogenesis	[21]

Rat MSCs	Hyaluronan	Unidirectional cyclic stretch bioreactor	Cell multilayer organization and invasion of the 3D mesh of the scaffold/muscle protein expression	[22]

HUVECs	Alginate	Multishear perfusion bioreactor	Expression of the intercellular adhesion molecule 1 (ICAM-1) and the phosphorylated endothelial nitric oxide synthase (eNOS)	[23]

Mouse endothelial cell line (MS-1)	Polycaprolactone porous scaffold	Shear perfusion bioreactor	Rapid endothelialization method to create preformed artificial vascular networks in scaffolds	[24]

Neonatal rat cardiac cells/rat endothelial cells	Cell sheets	Perfusion bioreactor	*In vivo* transplantation of a thick vascularized beating cardiac tissue	[25]

hESCs/hiPSCs/HUVECs/hMSCs	Collagen	Uniaxial stress bioreactor (FlexCell FX-4000T)	Cardiomyocyte and matrix fiber alignment/myofibrillogenesis and sarcomeric banding/increased vessel-like structures	[26]

## References

[1] Koudstaal S, Jansen of Lorkeers SJ, Gaetani R (2013). Concise review: heart regeneration and the role of cardiac stem cells. *Stem Cells Translational Medicine*.

[2] Pal SN, Kofidis T (2012). New cell therapies in cardiology. *Expert Review of Cardiovascular Therapy*.

[3] le Huu A, Paul A, Xu L, Prakash S, Shum-Tim D (2013). Recent advancements in tissue engineering for stem cell-based cardiac therapies. *Therapeutic Delivery*.

[4] Sanz-Ruiz R, Gutiérrez Ibañes E, Arranz AV, Fernández Santos ME, Fernández PL, Fernández-Avilés F (2010). Phases I-III clinical trials using adult stem cells. *Stem Cells International*.

[5] Mazhari R, Hare JM (2012). Translational findings from cardiovascular stem cell research. *Trends in Cardiovascular Medicine*.

[6] Zhang H, Chen H, Wang W, Wei Y, Hu S (2010). Cell survival and redistribution after transplantation into damaged myocardium. *Journal of Cellular and Molecular Medicine*.

[7] Karam JP, Muscari C, Montero-Menei CN (2012). Combining adult stem cells and polymeric devices for tissue engineering in infarcted myocardium. *Biomaterials*.

[8] Fiumana E, Pasquinelli G, Foroni L (2013). Localization of mesenchymal stem cells grafted with a hyaluronan-based scaffold in the infarcted heart. *Journal of Surgical Research*.

[9] Kannan RY, Salacinski HJ, Sales K, Butler P, Seifalian AM (2005). The roles of tissue engineering and vascularisation in the development of micro-vascular networks: a review. *Biomaterials*.

[10] Sng J, Lufkin T (2012). Emerging stem cell therapies: treatment, safety, and biology. *Stem Cells International*.

[11] Lesman A, Habib M, Caspi O (2010). Transplantation of a tissue-engineered human vascularized cardiac muscle. *Tissue Engineering A*.

[12] Stevens KR, Kreutziger KL, Dupras SK (2009). Physiological function and transplantation of scaffold-free and vascularized human cardiac muscle tissue. *Proceedings of the National Academy of Sciences of the United States of America*.

[13] Shepherd BR, Hoying JB, Williams SK (2007). Microvascular transplantation after acute myocardial infarction. *Tissue Engineering*.

[14] Dvir T, Kedem A, Ruvinov E (2009). Prevascularization of cardiac patch on the omentum improves its therapeutic outcome. *Proceedings of the National Academy of Sciences of the United States of America*.

[15] Chiu LL, Montgomery M, Liang Y, Liu H, Radisic M (2012). Perfusable branching microvessel bed for vascularization of engineered tissues. *Proceedings of the National Academy of Science of the United States of America*.

[16] Sasagawa T, Shimizu T, Sekiya S (2010). Design of prevascularized three-dimensional cell-dense tissues using a cell sheet stacking manipulation technology. *Biomaterials*.

[17] Moon JJ, Hahn MS, Kim I, Nsiah BA, West JL (2009). Micropatterning of poly(ethylene glycol) diacrylate hydrogels with biomolecules to regulate and guide endothelial morphogenesis. *Tissue Engineering A*.

[18] Liu Y, Markov DA, Wikswo JP, McCawley LJ (2011). Microfabricated scaffold-guided endothelial morphogenesis in three-dimensional culture. *Biomedical Microdevices*.

[19] Marsano A, Maidhof R, Luo J (2013). The effect of controlled expression of VEGF by transduced myoblasts in a cardiac patch on vascularization in a mouse model of myocardial infarction. *Biomaterials*.

[20] Sarig U, Au-Yeung GC, Wang Y (2012). Thick acellular heart extracellular matrix with inherent vasculature: a potential platform for myocardial tissue regeneration. *Tissue Engineering A*.

[21] Chung S, Sudo R, Zervantonakis IK, Rimchala T, Kamm RD (2009). Surface-treatment-induced three-dimensional capillary morphogenesis in a microfluidic platform. *Advanced Materials*.

[22] Govoni M, Lotti F, Biagiotti L (2012). An innovative stand-alone bioreactor for the highly reproducible transfer of cyclic mechanical stretch to stem cells cultured in a 3D scaffold. *Journal of Tissue Engineering and Regenerative Medicine*.

[23] Rotenberg MY, Ruvinov E, Armoza A, Cohen S (2012). A multi-shear perfusion bioreactor for investigating shear stress effects in endothelial cell constructs. *Lab on a Chip*.

[24] Kang TY, Hong JM, Kim BJ, Cha HJ, Cho DW (2013). Enhanced endothelialization for developing artificial vascular networks with a natural vessel mimicking the luminal surface in scaffolds. *Acta Biomaterialia*.

[25] Sekine H, Shimizu T, Sakaguchi K (2013). In vitro fabrication of functional three-dimensional tissues with perfusable blood vessels. *Nature Communication*.

[26] Tulloch NL, Muskheli V, Razumova MV (2011). Growth of engineered human myocardium with mechanical loading and vascular coculture. *Circulation Research*.

[27] Krawiec JT, Vorp DA (2012). Adult stem cell-based tissue engineered blood vessels: a review. *Biomaterials*.

[28] Muscari C, Bonafè F, Martin-Suarez S (2013). Restored perfusion and reduced inflammation in the infarcted heart after grafting stem cells with a hyaluronan-based scaffold. *Journal of Cellular and Molecular Medicine*.

[29] Xu S, Zhu J, Yu L, Fu G (2012). Endothelial progenitor cells: current development of their paracrine factors in cardiovascular therapy. *Journal of Cardiovascular Pharmacology*.

[30] Muscari C, Gamberini C, Basile I (2010). Comparison between culture conditions improving growth and differentiation of blood and bone marrow cells committed to the endothelial cell lineage. *Biological Procedures Online*.

[31] Resch T, Pircher A, Kähler CM, Pratschke J, Hilbe W (2012). Endothelial progenitor cells: current Issues on characterization and challenging clinical applications. *Stem Cell Reviews and Reports*.

[32] Baer PC, Geiger H (2012). Adipose-derived mesenchymal stromal/stem cells: tissue localization, characterization, and heterogeneity. *Stem Cells International*.

[33] Muscari C, Bonafè F, Fiumana E (2013). Comparison between stem cells harvested from wet and dry lipoaspirates. *Connective Tissue Research*.

[34] Huang S, Leung V, Peng S (2011). Developmental definition of MSCs: new insights into pending questions. *Cellular Reprogramming*.

[35] Si Y-L, Zhao Y-L, Hao H-J, Fu X-B, Han W-D (2011). MSCs: biological characteristics, clinical applications and their outstanding concerns. *Ageing Research Reviews*.

[36] Li M, Ikehara S (2013). Bone-marrow-derived mesenchymal stem cells for organ repair. *Stem Cells International*.

[37] Gwak S-J, Bhang SH, Yang HS (2009). In vitro cardiomyogenic differentiation of adipose-derived stromal cells using transforming growth factor-*β*1. *Cell Biochemistry and Function*.

[38] Pasini A, Bonafè F, Govoni M (2013). Epigenetic signature of early cardiac regulatory genes in native human adipose-derived stem cells. *Cell Biochemistry and Biophysics*.

[39] Boomsma RA, Geenen DL (2012). Mesenchymal stem cells secrete multiple cytokines that promote angiogenesis and have contrasting effects on chemotaxis and apoptosis. *PLoS One*.

[40] Penna C, Perrelli MG, Karam JP (2013). Pharmacologically active microcarriers influence VEGF-A effects on mesenchymal stem cell survival. *Journal of Cellular and Molecular Medicine*.

[41] Muscari C, Giordano E, Bonafè F, Govoni M, Pasini A, Guarnieri C (2013). Priming adult stem cells by hypoxic pretreatments for applications in regenerative medicine. *Journal of Biomedical Science*.

[42] Muscari C, Giordano E, Bonafè F, Govoni M, Pasini A, Guarnieri C (2013). Molecular mechanisms of ischemic preconditioning and postconditioning as putative therapeutic targets to reduce tumor survival and malignancy. *Medical Hypotheses*.

[43] Boheler KR, Joodi RN, Qiao H (2011). Embryonic stem cell-derived cardiomyocyte heterogeneity and the isolation of immature and committed cells for cardiac remodeling and regeneration. *Stem Cells International*.

[44] Okano H, Nakamura M, Yoshida K (2013). Steps toward safe cell therapy using induced pluripotent stem cells. *Circulation Research*.

[45] Anversa P, Kajstura J, Rota M, Leri A (2013). Regenerating new heart with stem cells. *Journal of Clinical Investigation*.

[46] Sukmana I (2012). Bioactive polymer scaffold for fabrication of vascularized engineering tissue. *Journal of Artificial Organs*.

[47] de Mel A, Jell G, Stevens MM, Seifalian AM (2008). Biofunctionalization of biomaterials for accelerated in situ endothelialization: a review. *Biomacromolecules*.

[48] Avci-Adali M, Paul A, Ziemer G, Wendel HP (2008). New strategies for in vivo tissue engineering by mimicry of homing factors for self-endothelialisation of blood contacting materials. *Biomaterials*.

[49] Zhao Y, Qiu F, Xu S, Yu L, Fu G (2011). Thymosin *β*4 activates integrin-linked kinase and decreases endothelial progenitor cells apoptosis under serum deprivation. *Journal of Cellular Physiology*.

[50] Mironov V, Visconti RP, Kasyanov V, Forgacs G, Drake CJ, Markwald RR (2009). Organ printing: tissue spheroids as building blocks. *Biomaterials*.

[51] Govoni M, Muscari C, Guarnieri C, Giordano E (2013). Mechanostimulation protocols for cardiac tissue engineering. *BioMed Research International*.

[52] Shimizu T, Sekine H, Yamato M, Okano T (2009). Cell sheet-based myocardial tissue engineering: new hope for damaged heart rescue. *Current Pharmaceutical Design*.

[53] Novosel EC, Kleinhans C, Kluger PJ (2011). Vascularization is the key challenge in tissue engineering. *Advanced Drug Delivery Reviews*.

[54] Stubbs SL, Crook JM, Morrison WA, Newcomb AE (2011). Toward clinical application of stem cells for cardiac regeneration. *Heart Lung and Circulation*.

